# Causality Between Irritable Bowel Syndrome and Suicide Attempt: A Mendelian Randomization Study

**DOI:** 10.1002/brb3.70513

**Published:** 2025-05-05

**Authors:** Zhen Deng, Kai Wang, Tianshu Hou

**Affiliations:** ^1^ Chengdu Integrated Traditional Chinese Medicine and Western Medicine Hospital Chengdu China; ^2^ Chengdu University of Traditional Chinese Medicine Chengdu China

**Keywords:** irritable bowel syndrome, IBS, suicide attempt, univariable Mendelian randomization, causal relationship, multivariable Mendelian randomization

## Abstract

**Background:**

Prior research has indicated a correlation between irritable bowel syndrome (IBS) and suicidal behavior. Nevertheless, it remains uncertain if this correlation implies causation.

**Methods:**

We used univariate and multivariate Mendelian randomization. The United Kingdom Biobank provided 53,400 European patients and 433,201 European controls for the IBS GWAS. The outcome variable was developed from a genome‐wide association analysis of 26,590 suicide attempt cases and 492,022 controls from the International Suicide Genetics Consortium. BioBank Finland GWAS data (9,771 cases and 402,410 controls) was used for SA validation. Primarily employing inverse variance weighting (IVW), we conducted the analysis to establish causality. MR‐Egger and weighted median were used as complementary methods to reinforce the robustness and validity of the results. We used the MRlap method to eliminate the effect of sample overlap. We also used a multivariable MR approach to control for the influence of potential confounders. Using a number of approaches, including the Cochran's Q test, the MR‐Egger intercept, and the MR‐PRESSO methodology, the study examined pleiotropy and heterogeneity.

**Results:**

We discovered evidence for an elevated risk of suicide attempt with IBS (OR = 1.67, 95% CI = 1.21–2.35, P = 5.52E–07). MRlap analyses similarly support this result. We got the same results with the validation data (OR = 1.19, 95% CI = 1.06–1.34, P = 2.46E–03). The relationships between the different sensitivity analysis approaches were similar, and there was no indication that outliers influenced these correlations. The independent causal impact of IBS on suicide attempts was maintained after controlling for anxiety, depression, and abdominal pain. In reverse MR, we found no causal link between suicide attempt and IBS.

**Conclusion:**

Our MR analysis indicates a causal relationship between IBS and suicide risk. Early detection and intervention in suicidal ideation in IBS patients reduces their suicide risk. More study is needed to understand the mechanisms that link IBS and suicidal behavior, which may alter or broaden therapy for specific individuals.

## Graphical abstract

Using univariable and multivariable Mendelian randomization (IVW, MR‐Egger, weighted median) on large European cohorts, we demonstrate a robust causal relationship between IBS and increased suicide attempt risk. These findings underscore the importance of early detection and targeted intervention in IBS patients to mitigate suicide risk.

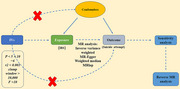

## 1 Introduction

Suicide is a significant worldwide issue in public health. Based on survey data from the World Health Organization (WHO), more than 800,000 individuals die by suicide each year, with suicide attempts (SA) multiple times greater than suicide fatalities (Fazel and Runeson 2020, Bostwick et al. 2016). SA is defined as self‐injurious conduct with the aim of dying, and the lifetime prevalence of SA in individuals worldwide is estimated to be between 0.5 and 5% (Nock et al. 2008). This not only increases the risk of personal handicap and lower quality of life but also places a significant strain on families and society as a whole. Suicide is a complicated outcome of the combination of genetic, biochemical, psychological, environmental, and social variables, making it an important subject of research (Turecki et al. 2019).

Irritable bowel syndrome (IBS) is a common and potentially severe functional bowel illness marked by recurring abdominal discomfort or changes in bowel habits (Chey et al. 2015), resulting in a significant social cost (Ballou and Keefer 2017, Corsetti and Whorwell 2017, Mearin et al. 2014, Böhn et al. 2013, Jung et al. 2014). It has an estimated global frequency of 11.2%, ranging from 1.1 to 45% in the general population (Lovell and Ford 2012), and it can impose a large financial burden on healthcare systems and society (Corsetti and Whorwell 2017, Canavan et al. 2014). Approximately 40%‐60% of IBS patients have concomitant mental illnesses (Drossman et al. 2002), and 38% consider suicide due to their symptoms (Miller et al. 2004). Furthermore, current evidence indicates that the development of IBS increases the likelihood of suicide ideation, and this link exists regardless of co‐morbid depression (Jiang et al. 2019).

Although previous research has demonstrated higher suicidal thoughts in people with IBS and associated symptoms (Miller et al. 2004, Ballou et al. 2015, Deutsch et al. 2021, Jiang et al. 2019, Minocha et al. 2010, Spiegel et al. 2007, Tarar et al. 2023), it is unclear if there is a causative link between IBS and suicide risk. Furthermore, most research varies in terms of study design, patient demographics, and terminology, owing to methodological constraints (Spiegel et al. 2007). It is crucial to determine whether there is a causal relationship between irritable bowel syndrome and suicide risk. This information is essential for suicide prevention in both somatic and psychiatric care. Effective interventions can only be developed if the risk factors being targeted are causally linked to the desired outcome rather than simply being correlated.

Quasi‐experimental techniques, such as Mendelian randomization, can be employed to enhance causal inferences made from observational data in the context of IBS and suicide. This is because conducting randomized studies to establish causation in this area is both unethical and impractical. Within an instrumental variable (IV) framework, MR makes use of genetic data (Sekula et al. 2016). By substituting standard observational multivariate regression with MR, one may circumvent prevalent problems in observational research, such as confounding factors and reverse causality bias (Davey Smith et al. 2020). Utilizing data from extensive genome‐wide association studies (GWASs), this research used Mendelian randomization to investigate the causal link between IBS and suicide attempts.

## 2 Methods

### 2.1 Study Design

This study used a bidirectional MR design to find possible causal links and avoid false negative causation. As an expansion of two‐sample MR analysis, bidirectional MR analysis requires the measurement of both the outcome and exposure factors using IVs, and it employs both methods for genetic prediction‐based causal analysis (Davey Smith and Hemani 2014).

**FIGURE 1:**
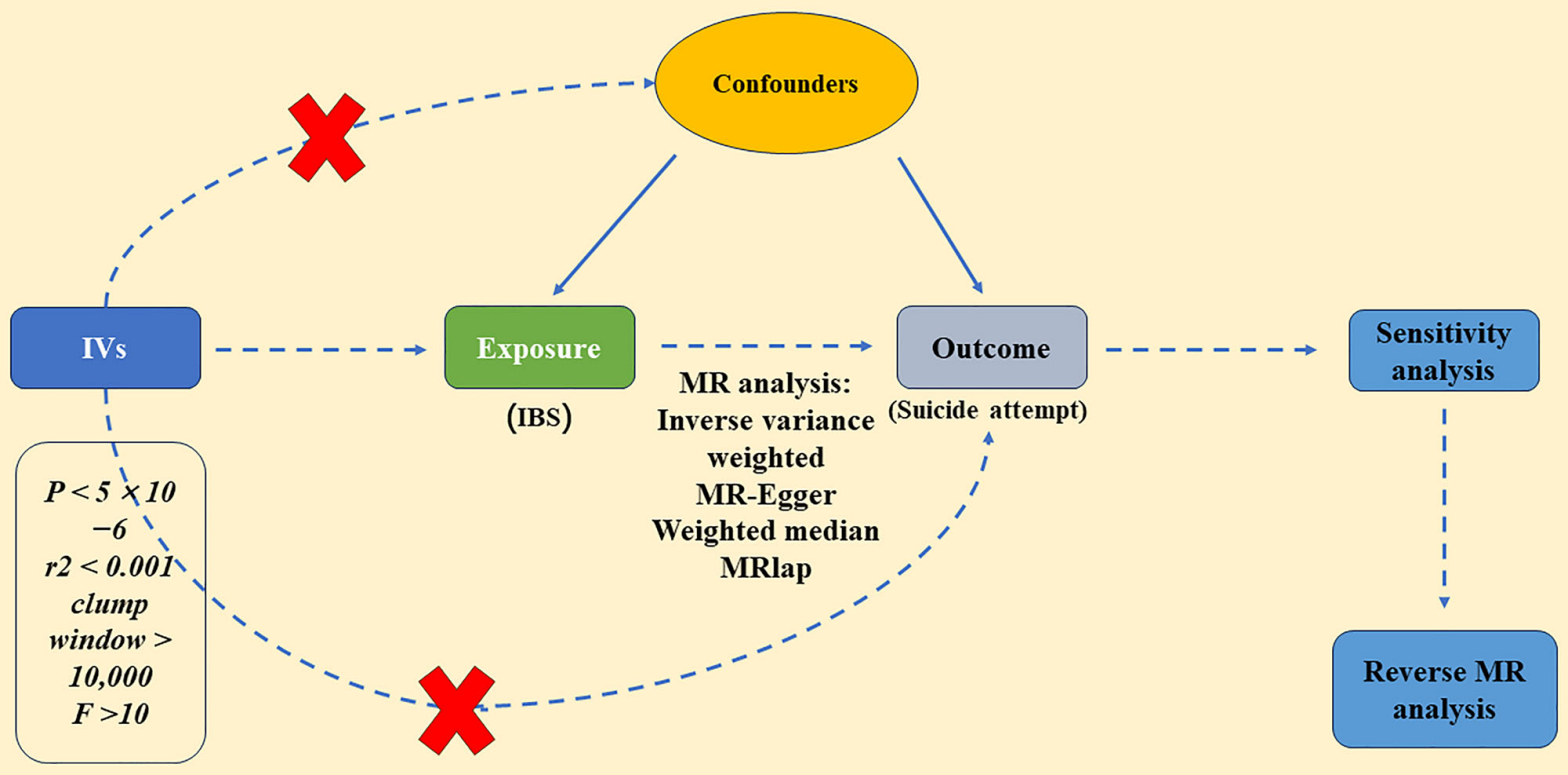
The study design of our MR investigation. MR, Mendelian randomization; IVs, instrumental variables; IBS, irritable bowel syndrome.

In order to guarantee effective causal reasoning in MR research, three prerequisites must be fulfilled: (Fazel and Runeson 2020) genetic IVs with strong associations to exposure; (Bostwick et al. 2016) genetic IVs are independent of potential confounding variables; and (Nock et al. 2008) specific genetic IVs are influenced by exposures while other factors are not (Lawlor 2016). Figure 1 depicts a schematic for an MR. As ethical approval and informed permission were already obtained for each of the initial investigations, there was no need to undertake any additional ethical evaluations.

### 2.2 Data Sources

#### 2.2.1 Exposure

The IBS GWAS comprised a sample of 53,400 European individuals with the condition and 433,201 European individuals without it, obtained from the United Kingdom Biobank (UKB) (Eijsbouts et al. 2021). IBS cases must meet at least one of the following four criteria (Ford et al. 2020): The Rome III symptom criteria for the Digestive Health Questionnaire (DHQ) are considered fulfilled if the symptoms of IBS cannot be sufficiently explained by any other diagnosis (Vasant et al. 2021). The DHQ “self‐report” method involves individuals responding affirmatively when asked if they have ever been diagnosed with IBS (Stern 2021). Unprompted “self‐report”: spontaneously stated an IBS diagnosis when asked whether they had been advised of any major medical disorders (Song et al. 2021); International Code of Disease Version 10 (ICD‐10).

#### 2.2.2 Outcome

The major data on suicide attempts came from a GWAS comprising 26,590 suicide attempt (SA) cases and 492,022 controls conducted by the International Suicide Genetics Consortium (ISGC) (Mullins et al. 2022). This covers 14 cohorts of European ancestry. The examples included individuals who attempted suicide or completed suicide without deadly consequences. A nonfatal suicide attempt was described as a lifelong act of purposeful self‐harm with the aim of dying. Individuals who merely expressed suicidal ideation or nonsuicidal self‐injurious conduct were excluded as cases.

The FinnGen Consortium is a continuous genetic research initiative that combines genetic information from the Finnish Biobank with information from the Finnish Health Registry's digital health records (FinnGen, 1985). We utilized GWAS data from Finland (R10) on suicide or intentional self‐harm incidents as validation data. The endpoint of these incidents was determined based on whether an individual was hospitalized or died because of suicide or deliberate self‐harm (FinnGen, 1985). The sample consisted of 9,771 cases and 402,410 controls.

### 2.3 Instrumental Selection

Initially, the single nucleotide polymorphisms (SNPs) linked to both the exposure and result have achieved statistical significance at the genome‐wide level (*P* <  5 × 10^−8^). Nevertheless, because there are only a few IVs that meet the criteria (*P*  <  5 × 10^−8^), a broader criterion (*P*  <  5 × 10^−6^) has been used (Davey Smith and Hemani 2014). Second, in order to satisfy the MR hypothesis, the presence of strong linkage disequilibrium (LD) may introduce bias in the results. In order to rule out the potential for linkage disequilibrium between these SNPs (clump window > 10,000 kb, r^2^ < 0.001), we used a reference collection of 1,000 genomes derived from a European population. Palindromic SNPs were also manually eliminated. Following these procedures, the remaining SNPs were employed as instrument variables. In order to eliminate the possibility of pleiotropic effects, we conducted a search for secondary phenotypes of each single nucleotide polymorphism (SNP) in the NHGRI‐EBI Catalog (https://www.ebi.ac.uk/gwas/). SNPs associated with the trait of interest were removed, and the remaining SNPs were utilized for subsequent research. In addition, we assessed the statistical effectiveness of each SNP using the F‐statistic (F = β^2^/se^2^) (Saadh et al. 2023) and eliminated any low statistical effectiveness SNPs to minimize slight instrumental bias (F > 10).

### 2.4 Univariable Mendelian Randomization Analysis

We employed many complementary methodologies, including weighted median, MR‐Egger, and IVW. Using the IVW technique, we assessed the causal connection between IBS and suicide attempt in our major studies. We believed that the IVW method was the most effective method for estimating causal effects when handling exposures to many IVs (Burgess et al. 2019). As a result, the IVW approach was our primary technique of analysis for MR. When IVs showed notable heterogeneity (*P* < 0.05), random‐effect IVW was used to estimate the MR effect size. In the absence of it, fixed‐effect IVW was used (Greco et al. 2015).

### 2.5 Multivariable Mendelian Randomization Analysis

While several studies have indeed established a link between IBS and suicide attempts, they have not successfully eliminated the impact of confounding variables. The confounding factors mostly consist of depression (Miller et al. 2004, Whitehead et al. 2002), anxiety (Whitehead et al. 2002), and abdominal pain (Spiegel et al. 2007). Several academics propose that these variables operate as mediators in the relationship between IBS and suicide attempts (Spiegel et al. 2007). To evaluate the independent causal connection between IBS and suicide attempts, we performed a multivariate MR analysis.

### 2.6 Sensitivity Analysis

We conducted numerous sensitivity studies to evaluate the results' robustness. The heterogeneity of the IVW was initially assessed using the Cochran's Q test (Bowden et al. 2016), while funnel plots were utilized to visually present the outcomes. Additionally, in order to investigate pleiotropy, the MR‐Egger intercept test results were presented using scatterplots (Bowden et al. 2015). The MR‐PRESSO test is designed to identify outliers and determine the causal effects following the removal of the related outliers. Additionally, it is used to evaluate the existence of horizontal pleiotropy (Verbanck et al. 2018). In order to ascertain whether any particular SNP had a substantial effect on the outcomes, we implemented a leave‐one‐out analysis.

### 2.7 MRlap Analysis

Because some of the cohorts in the attempted suicide (ISGC) sample were from UKB, MR analyses can be affected by sample overlap and produce false positives. To mitigate any potential bias arising from our inability to directly ascertain the sample overlap rate (Mounier and Kutalik 2023), we employed the MRlap function to adjust the IVW findings. One can have confidence in the IVW‐MR calculations if the difference between the observed and adjusted effects is not statistically significant (*P* > 0.05). On the other hand, if we see a notable disparity (*P* < 0.05), we should give priority to the adjusted effect, which stays unaltered by sample overlap.

RStudio (version 4.2.2) was employed in conjunction with the packages “TwoSampleMR” (version 0.5.11), Mrlap (version 0.0.3.2), and MRPRESSO'' (version 1.0) to conduct the comprehensive analysis.

## 3 Results

### 3.1 Causal Effect of IBS on Suicide Attempt

**FIGURE 2:**
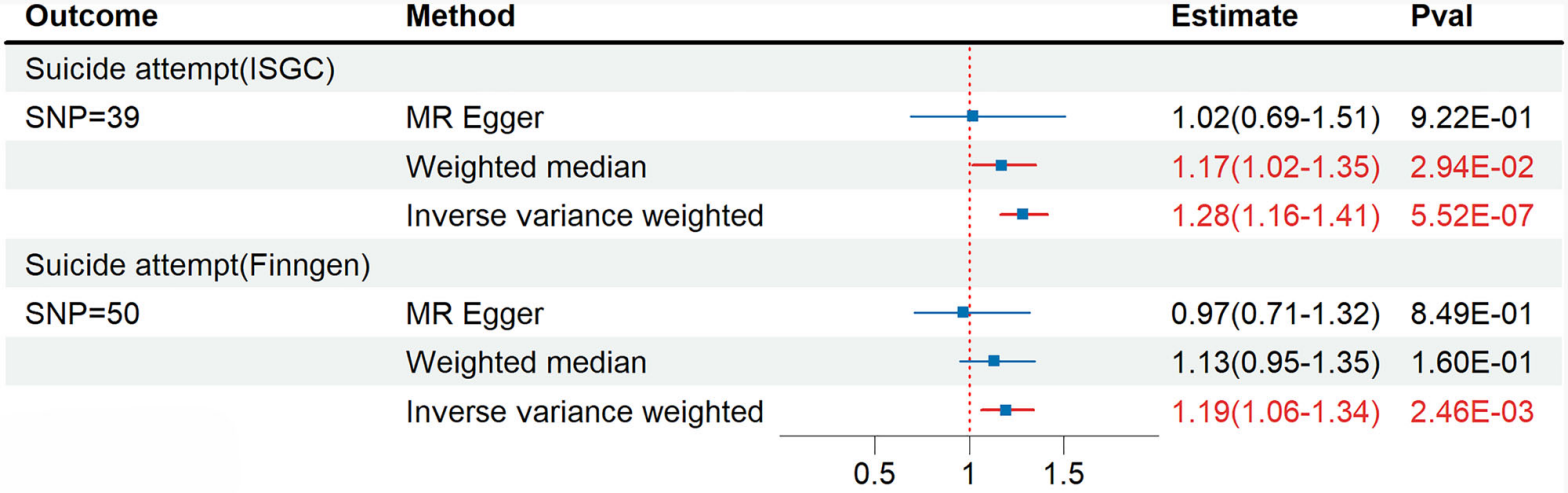
MR analysis for IBS on suicide attempt; estimate, odds ratio estimate.

Figure 2 provides evidence of a correlation between IBS and suicide attempts. Applying the IVW method, we found an odds ratio (OR) of 1.28 (95% confidence interval [CI], 1.16 ‐ 1.41), indicating a 28% rise in the likelihood of a suicide attempt for every additional unit increase in the IBS measure unit. The heterogeneity of this estimate was minimal, as indicated by the Q statistic (Table 1). The Egger intercept provided no indication of any imbalanced horizontal pleiotropy (9.36E‐03; *P* = 2.44E‐01). The primary analysis was supported by the results of the sensitivity analyses (Table 1). Outlier SNPs were not found by either the MR‐PRESSO or the leave‐one‐out analysis. The same evidence (IVW, OR = 1.19, 95% CI = 1.16‐1.41, *P* = 2.46E‐03) is obtained in the validation queue. This implies that the data supporting a link between IBS and suicide attempts is trustworthy. The results of other sensitivity analyses can be found in the supplementary material.

**TABLE 1:** Sensitivity analysis of the associations between IBS and suicide risk.

Exposures	Outcomes	Heterogeneity test				Pleiotropy test		
		MR‐Egger		IVW		MR‐Egger intercept		MR‐PRESSO
		Q	pval	Q	pval	Intercept	pval	Global test pval
IBS	Suicide attempt(ISGC)	39.93	3.41E‐01	41.44	3.23E‐01	9.36E‐03	2.44E‐01	3.73E‐01
IBS	Suicide attempt(Finngen)	47.60	4.89E‐01	49.60	4.49E‐01	9.95E‐03	1.64E‐01	4.68E‐01
Suicide attempt(ISGC)	IBS	36.18	3.22E‐01	36.19	3.67E‐01	−5.22E‐04	9.28E‐01	3.51E‐01
Suicide attempt(Finngen)	IBS	25.17	6.19E‐01	27.29	5.56E‐01	5.68E‐03	1.57E‐01	5.47E‐01

### 3.2 Causal Effect of Suicide Attempt on IBS

**FIGURE 3:**
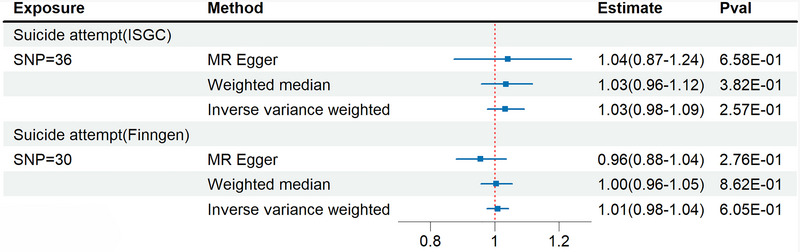
MR analysis for suicide attempt on IBS; estimate, odds ratio estimate.

In the reverse analysis (Figure 3), our MR analyses demonstrated that there is no correlation between IBS and suicide attempts (IVW: OR = 1.03, 95% CI = 0.98‐1.09, *P* = 2.57E‐01). Our validation queue analysis yielded the same outcome (IVW: OR = 1.01, 95% CI = 0.98‐1.04, *P* = 6.05E‐01). The preliminary analysis (Table 1) is substantiated by all sensitivity analysis results, and the Supplementary Material contains the results of additional sensitivity analyses.

### 3.3 Sensitivity Analysis

In our sensitivity analysis (Table 1), we performed comprehensive heterogeneity tests using both the MR‐Egger and IVW approaches, where the Cochran's Q test yielded non‐significant results (p > 0.05), indicating a consistent effect across individual SNPs and affirming the reliability of our genetic instruments. In parallel, we assessed potential pleiotropy through the MR‐Egger intercept test and the MR‐PRESSO global test; the MR‐Egger intercept did not reach statistical significance (p > 0.05), and MR‐PRESSO did not detect any outliers, collectively confirming that there is no evidence of horizontal pleiotropy biasing our results. These rigorous sensitivity analyses substantiate that our Mendelian randomization estimates are robust and reliable, thus providing strong support for the conclusion that IBS has a causal effect on an increased risk of suicide attempt.

### 3.4 Multivariable MR Results

After adjusting for depression, anxiety, and abdominal pain, the multivariate MR analysis yielded results that were in agreement with the findings of the univariate MR studies (Table 2). This evidence suggests that the causal relationship between IBS and suicide attempts is independent of depression, anxiety, and abdominal pain.

**TABLE 2:** Multivariable MR results for the causal relationship of IBS with suicide attempt.

Exposure	Adjustment	Outcome	Beta	OR (95%CI)	*P*
IBS	Depression, anxiety disorders, Stomach or abdominal pain	Suicide attempt(ISGC)	0.19	1.21(1.07‐1.37)	2.29E‐03

Abbreviations: CI, confidence interval.; OR, odds ratio.

### 3.5 MRlap Analysis

The results of the MRlap analysis are shown in Table 3. The IVW method's robustness is confirmed by the fact that the data corrected by MRlap agreed with the findings of the initial MR analysis. Similarly, it was shown that sample overlap had no effect on the causal connection between IBS and suicide attempts.

**TABLE 3:** Results of MRlap analysis.

Exposure	Outcome	Corrected_effect	Corrected_effect_p	Difference_p
IBS	Suicide attempt (ISGC)	2.82E‐01	1.79E‐08	2.83E‐07

## 4 Discussion

In this MR study, we looked at the bidirectional causal relationship between IBS and suicide attempts. This study is a first effort to look at the possible cause‐and‐effect link between IBS and suicide attempts. Our findings support earlier studies showing a link between IBS and a higher incidence of suicidal thoughts. Nonetheless, there was no discernible causal relationship between IBS and suicide attempters.

In this study, we conducted a bidirectional MR analysis using genome‐wide data for IBS and SA. Since the IBS and SA data were both derived from multiple large cohorts, particularly from the United Kingdom Biobank and the ISGC, there is potential for sample overlap. Sample overlap can introduce several biases: first, some individuals may be included in both studies, leading to statistical duplication, which could affect the accuracy of genetic associations. This is especially problematic when making causal inferences across datasets, as overlapping samples could bias the estimation of genetic effects. Secondly, sample overlap could increase statistical power in the analysis, thereby overestimating the causal relationship or genetic correlation between IBS and SA.

To address this issue, we implemented several measures to minimize the impact of sample overlap on our analysis results. First, we used independent SA data from Finland as a validation cohort. Since this cohort does not overlap with the IBS sample, it effectively tests the potential impact of sample overlap on our primary analysis results. Second, in the main SA analysis, we employed the MRlap method, which independently applies genetic instruments between the exposure and outcome variables, reducing the potential interference caused by sample overlap. The MRlap method significantly enhanced the accuracy and reliability of our analysis, thereby mitigating the impact of sample overlap to the greatest extent possible.

Research has indicated that an IBS diagnosis is linked to a lower quality of life, job loss, ineffective time management, non‐adherence to medicine, and higher rates of readmission to hospitals. According to certain research, it may be linked to mental health issues, including anxiety. 50–60% of IBS patients have been shown to have significant psychological issues (Hausteiner‐Wiehle and Henningsen 2014, Farzaneh et al. 2012). Furthermore, there is a significant comorbidity of various medical and psychiatric illnesses with IBS, which contributes to its total burden. Comorbid extra‐intestinal symptoms and illnesses, such as fibromyalgia (Sperber et al. 1999), back pain (Smith et al. 2008), urogenital issues (Guo et al. 2010), and sleep issues (Elsenbruch et al. 1999), affect around 65% of individuals with IBS (Riedl et al. 2008). Additionally, concomitant mental diagnoses such as anxiety disorders, depression, and post‐traumatic stress disorder are reported by 40%–60% of IBS patients, compared to 20% of the general population (Drossman et al. 2002, Cohen et al. 2006). IBS patients in tertiary care settings have considered suicide up to 38% of the time due to symptoms (Miller et al. 2004), and they are also more likely to report having a terrible quality of life (Koloski et al. 2000).

One of the possible causes is the nature of IBS and how it progresses to lower patients' quality of life. According to Polish research, IBS patients' quality of life (QOL) was reportedly poorer than that of the control group (Kopczyńska et al. 2018). A small number of studies found that the QOL of patients with IBS was notably worse than that of individuals with other chronic conditions, such as end‐stage renal disease, inflammatory bowel disease, liver disease, CHF, diabetes mellitus, and chronic pancreatitis (Gralnek et al. 2004, El‐Serag et al. 2002). Such conditions may result in financial difficulties and psychological stress, which starts a vicious cycle that feeds back on itself and eventually results in worry and despair.

Another hypothesis for the link between IBS and mental disorders is the “brain‐gut” axis. In IBS patients with mental problems, immunological, intestinal, autonomic, and microbiome changes have been described (Midenfjord et al. 2019). Brain‐gut axis dysfunction, a bidirectional neurological link connecting the brain and digestive system, may cause symptoms of IBS, according to previous psychophysiological and neuroimaging research. Concurrently, dysregulation of the gut microbiota and its subsequent impact on the gut‐brain axis represents another critical mechanism linking IBS to increased suicide risk (Ng et al. 2023). Multiple studies have demonstrated that IBS patients exhibit significant alterations in gut microbial composition, which compromise intestinal barrier function and facilitate the translocation of endotoxins and proinflammatory mediators into systemic circulation (Zhou et al. 2018). This microbial imbalance may disrupt neuroimmune and neuroendocrine communication, contributing to cognitive and mood disorders that elevate the propensity for suicidal behavior. Moreover, the gut microbiota produces neuroactive substances that directly influence neural transmission, and its dysbiosis could lead to aberrant communication between brain regions, establishing a hyperexcitable neural network that correlates with the observed increase in suicide risk among IBS patients (Zhou et al. 2023, Tyagi et al. 2020). According to this view, IBS symptoms cause anxiety and depression, and psychiatric illnesses or psychological variables may worsen IBS symptoms (Fadgyas‐Stanculete et al. 2014, Longarzo et al. 2017). Chronic disorders like IBS can destabilize quality of life and cause stress, work impairment, and cost. Thus, IBS treatment and mood disorder diagnosis improve quality of life. Research has demonstrated the effectiveness of antidepressants and psychological treatments in treating individuals with irritable bowel syndrome (IBS), similar to how they are effective in treating mental conditions (Ford et al. 2019, Laird et al. 2017).

Chronic pain, chronic abdominal discomfort, and IBS are all linked to higher rates of mental co‐morbidity (Whitehead et al. 2002, Demyttenaere et al. 2007, Nakao and Yano 2006). Chronic pain patients are at a much higher risk of depression and anxiety, independent of the pain site (Fishbain et al. 1997, McWilliams et al. 2003, Edwards et al. 2006). IBS patients often endure chronic abdominal pain and discomfort that can trigger persistent stress responses, leading to sustained activation of the hypothalamic‐pituitary‐adrenal (HPA) axis and subsequent neuroendocrine and immune dysregulation (Hubbard et al. 2011). This chronic stress may result in the prolonged release of proinflammatory cytokines, such as IL‐6 and TNF‐α (Brzozowski et al. 2016), which in turn can induce neuroinflammation and alter neurotransmitter synthesis and release—particularly serotonin—thereby exacerbating symptoms of depression and anxiety (Leite et al. 2024). These psychological disturbances not only diminish patients' quality of life but may also disrupt neural circuits involved in emotion regulation, thus heightening the risk of suicidal behavior (Ressler 2020). Furthermore, the enduring inflammatory state may impair neuronal plasticity and synaptic function, further undermining the brain's capacity to manage stress and emotion (McIntyre et al. 2020), which supports the biological plausibility of a causal link between IBS and suicide attempt (Miller et al. 2004). There have been more than 20 studies on the connection between IBS and mental diseases. Whitehead et al.’s systematic review evaluated these studies critically (Whitehead et al. 2002). According to a comprehensive study, at least half of community‐based IBS patients suffer from one or more mental health issues (Spiegel et al. 2007). IBS is linked to a significantly higher burden of mental co‐morbidity than matched controls without IBS, according to Whitehead et al.’s findings, which hold true across many studies in various populations. Even after adjusting for mental co‐morbidity, a number of the evaluated studies revealed that suicide behavior rates were elevated in patients with persistent abdominal pain (Smith et al. 2004, Magni et al. 1998). This suggests that suicide behavior may be significantly influenced by psychological or psychosocial problems unique to pain, in addition to depression and anxiety.

Our study primarily involved a European population, which naturally raises concerns about the generalizability of our findings to other ethnic groups and genders. Although the homogeneity of a European cohort can enhance statistical power and minimize confounding due to population stratification, differences in genetic architecture, environmental exposures, and lifestyle factors across ethnicities may result in variable manifestations of IBS and its associated outcomes. For instance, gene‐environment interactions that drive the causal relationship observed in our study may differ in non‐European populations, where distinct genetic backgrounds and environmental contexts prevail. Similarly, gender‐specific factors such as hormonal differences, which can influence both the prevalence and the clinical presentation of IBS, may modulate the impact on mental health outcomes differently than in our primarily European sample.

Moreover, while our findings provide a robust methodological framework and foundational insights, the limitation regarding sample diversity suggests that caution is warranted when extrapolating these results to broader populations. Future studies should aim to replicate our analyses in more diverse cohorts to confirm the consistency of these causal relationships across various ethnic groups and between genders. Such investigations would not only enhance the external validity of the current findings but also help identify potential modifiers of the association between IBS and adverse outcomes like suicide attempts. By addressing these issues in subsequent research, the current limitation can be mitigated, ultimately facilitating a more comprehensive understanding of IBS's impact on different demographic groups.

This is the first investigation into the possible causal relationship between IBS and suicide attempt. Biases may still exist even after confounding variables have been adjusted in certain previous cross‐sectional research. In addition, we utilized genetic data from a large sample, which increased the accuracy of our conclusions and decreased the impact of any variables that may skew them. Moreover, multivariate MR was applied. Even after correcting for the impact of anxiety, depression, and abdominal pain, there was still a causal link between IBS and suicide attempt.

Our study acknowledges that GWAS data can be subject to measurement errors and phenotypic discrepancies, which may arise from differences in diagnostic criteria, self‐reporting biases, or variability in phenotype definitions across cohorts. Such inconsistencies could lead to misclassification, potentially attenuating the observed associations between IBS and suicide attempts by diluting the true genetic effect sizes. Although these issues might introduce some level of uncertainty in our effect estimates, our extensive sensitivity analyses—including MR‐Egger and MR‐PRESSO tests—consistently support the robustness of our findings. Nonetheless, we recognize that standardizing phenotypic measurements in future studies and expanding the scope to include diverse populations could further solidify the causal inference and improve the generalizability of our conclusions.

This work on bidirectional MR has some limitations. At first, the majority of the GWAS data that we used came from people who were European in origin. Therefore, it's possible that the research's conclusions cannot be applied to other demographic groups. Secondly, the lack of gender‐specific GWASs prevented us from classifying analyses by gender. In a similar vein, age's impact could not be investigated in detail. Lastly, the GWASs from large populations that were employed in this investigation may have had measurement errors or been diverse in terms of phenotypic parameters (such as sex and age distributions). For example, tracking suicide attempts based on medical information obtained from hospitals misses instances in which care was never given.

## 5 Conclusion

To summarize, the MR analysis offers data that supports the causal link between IBS and suicide attempt. Our research suggests that individuals with IBS have a higher likelihood of attempting suicide. The correlation between suicide attempt and IBS was not statistically significant.

The study is clinically significant. First, increasing knowledge about this correlation may enhance the identification of those who possess suicidal thoughts but have not inquired about them. Second, prompt referral and treatment might result from early detection of precursors to attempted suicide, such as continued self‐harming behavior or suicidal thoughts. Furthermore, comprehending the mechanism that connects IBS and suicidal behavior has the potential to modify or broaden treatment strategies for particular individuals.

## Author Contributions

**Zhen Deng**: conceptualization, investigation, methodology, validation, writing – review and editing, writing – original draft, visualization, supervision, data curation. **Kai Wang**: visualization, data curation. **Tianshu Hou**: writing – review and editing, supervision.

## Ethics Statement

We derived all of the data for our MR study from summary figures that were previously released to the public. Thus, the investigation can proceed without obtaining ethical clearance or patient consent.

## Conflicts of Interest

The authors declare no conflicts of interest.

### Peer Review

The peer review history for this article is available at https://publons.com/publon/10.1002/brb3.70513

## Supporting information



Supporting Information

## Data Availability

The corresponding author can provide the datasets used and/or analyzed in this study upon reasonable request.

## References

[brb370513-bib-0001] Ballou, S. , A. Bedell , and L. Keefer . 2015. “Psychosocial Impact of Irritable Bowel Syndrome: A Brief Review.” World Journal of Gastrointestinal Pathophysiology 6, no. 4: 120–123. 10.4291/wjgp.v6.i4.120. 26600969 PMC4644875

[brb370513-bib-0002] Ballou, S. , and L. Keefer . 2017. “The Impact of Irritable Bowel Syndrome on Daily Functioning: Characterizing and Understanding Daily Consequences of Ibs.” Neurogastroenterology and Motility 29, no. 4: e12982. 10.1111/nmo.12982. PMC5367953 27781332

[brb370513-bib-0003] Böhn, L. , S. Störsrud , H. Törnblom , U. Bengtsson , and M. Simrén . 2013. “Self‐Reported Food‐Related Gastrointestinal Symptoms in Ibs Are Common and Associated With More Severe Symptoms and Reduced Quality of Life.” The American Journal of Gastroenterology 108, no. 5: 634–641. 10.1038/ajg.2013.105. 23644955

[brb370513-bib-0004] Bostwick, J. M. , C. Pabbati , J. R. Geske , and A. J. McKean . 2016. “Suicide Attempt as a Risk Factor for Completed Suicide: Even More Lethal Than We Knew.” The American Journal of Psychiatry 173, no. 11: 1094–1100. 10.1176/appi.ajp.2016.15070854. 27523496 PMC5510596

[brb370513-bib-0005] Bowden, J. , G. Davey Smith , and S. Burgess . 2015. “Mendelian Randomization With Invalid Instruments: Effect Estimation and Bias Detection Through Egger Regression.” International Journal of Epidemiology 44, no. 2: 512–525. 10.1093/ije/dyv080. 26050253 PMC4469799

[brb370513-bib-0006] Bowden, J. , G. Davey Smith , P. C. Haycock , and S. Burgess . 2016. “Consistent Estimation in Mendelian Randomization With Some Invalid Instruments Using a Weighted Median Estimator.” Genetic Epidemiology 40, no. 4: 304–314. 10.1002/gepi.21965. 27061298 PMC4849733

[brb370513-bib-0007] Brzozowski, B. , A. Mazur‐Bialy , R. Pajdo , et al. 2016. “Mechanisms by Which Stress Affects the Experimental and Clinical Inflammatory Bowel Disease (Ibd): Role of Brain‐Gut Axis.” Current Neuropharmacology 14, no. 8: 892–900. 10.2174/1570159x14666160404124127. 27040468 PMC5333596

[brb370513-bib-0008] Burgess, S. , G. Davey Smith , N. M. Davies , et al. 2019. “Guidelines for Performing Mendelian Randomization Investigations: Update for Summer 2023.” Wellcome Open Research 4: 186. 10.12688/wellcomeopenres.15555.3. 32760811 PMC7384151

[brb370513-bib-0009] Canavan, C. , J. West , and T. Card . 2014. “Review Article: The Economic Impact of the Irritable Bowel Syndrome.” Alimentary Pharmacology & Therapeutics 40, no. 9: 1023–1034. 10.1111/apt.12938. 25199904

[brb370513-bib-0010] Chey, W. D. , J. Kurlander , and S. Eswaran . 2015. “Irritable Bowel Syndrome: A Clinical Review.” Jama 313, no. 9: 949–958. 10.1001/jama.2015.0954. 25734736

[brb370513-bib-0011] Cohen, H. , A. Jotkowitz , D. Buskila , et al. 2006. “Post‐Traumatic Stress Disorder and Other Co‐Morbidities in a Sample Population of Patients With Irritable Bowel Syndrome.” European Journal of Internal Medicine 17, no. 8: 567–571. 10.1016/j.ejim.2006.07.011. 17142176

[brb370513-bib-0012] Corsetti, M. , and P. Whorwell . 2017. “The Global Impact of Ibs: Time to Think About Ibs‐Specific Models of Care?” Therapeutic Advances in Gastroenterology 10, no. 9: 727–736. 10.1177/1756283x17718677. 28932273 PMC5598808

[brb370513-bib-0013] Davey Smith, G. , and G. Hemani . 2014. “Mendelian Randomization: Genetic Anchors for Causal Inference in Epidemiological Studies.” Human Molecular Genetics 23, no. R1: R89–R98. 10.1093/hmg/ddu328. 25064373 PMC4170722

[brb370513-bib-0014] Davey Smith, G. , M. V. Holmes , N. M. Davies , and S. Ebrahim . 2020. “Mendel's Laws, Mendelian Randomization and Causal Inference in Observational Data: Substantive and Nomenclatural Issues.” European Journal of Epidemiology 35, no. 2: 99–111. 10.1007/s10654-020-00622-7. 32207040 PMC7125255

[brb370513-bib-0015] Demyttenaere, K. , R. Bruffaerts , S. Lee , et al. 2007. “Mental Disorders Among Persons With Chronic Back or Neck Pain: Results From the World Mental Health Surveys.” Pain 129, no. 3: 332–342. 10.1016/j.pain.2007.01.022. 17350169

[brb370513-bib-0016] Deutsch, D. , M. Bouchoucha , G. Devroede , J. J. Raynaud , J. M. Sabate , and R. Benamouzig . 2021. “Functional Gastrointestinal Disorders as Predictors of Suicidal Ideation. An Observational Study.” European Journal of Gastroenterology & Hepatology 33, no. Suppl 1: e758–e765. 10.1097/meg.0000000000002245. 34231520

[brb370513-bib-0017] Drossman, D. A. , M. Camilleri , E. A. Mayer , and W. E. Whitehead . 2002. “Aga Technical Review on Irritable Bowel Syndrome.” Gastroenterology 123, no. 6: 2108–2131. 10.1053/gast.2002.37095. 12454866

[brb370513-bib-0018] Edwards, R. R. , M. T. Smith , I. Kudel , and J. Haythornthwaite . 2006. “Pain‐Related Catastrophizing as a Risk Factor for Suicidal Ideation in Chronic Pain.” Pain 126, no. 1‐3: 272–279. 10.1016/j.pain.2006.07.004. 16926068

[brb370513-bib-0019] Eijsbouts, C. , T. Zheng , N. A. Kennedy , et al. 2021. “Genome‐Wide Analysis of 53,400 People With Irritable Bowel Syndrome Highlights Shared Genetic Pathways With Mood and Anxiety Disorders.” Nature Genetics 53, no. 11: 1543–1552. 10.1038/s41588-021-00950-8. 34741163 PMC8571093

[brb370513-bib-0020] Elsenbruch, S. , M. J. Harnish , and W. C. Orr . 1999. “Subjective and Objective Sleep Quality in Irritable Bowel Syndrome.” The American Journal of Gastroenterology 94, no. 9: 2447–2452. 10.1111/j.1572-0241.1999.01374.x. 10484007

[brb370513-bib-0021] El‐Serag, H. B. , K. Olden , and D. Bjorkman . 2002. “Health‐Related Quality of Life Among Persons With Irritable Bowel Syndrome: A Systematic Review.” Alimentary Pharmacology & Therapeutics 16, no. 6: 1171–1185. 10.1046/j.1365-2036.2002.01290.x. 12030961

[brb370513-bib-0022] Fadgyas‐Stanculete, M. , A. M. Buga , A. Popa‐Wagner , and D. L. Dumitrascu . 2014. “The Relationship Between Irritable Bowel Syndrome and Psychiatric Disorders: From Molecular Changes to Clinical Manifestations.” Journal of Molecular Psychiatry 2, no. 1: 4. 10.1186/2049-9256-2-4. 25408914 PMC4223878

[brb370513-bib-0023] Farzaneh, N. , M. Ghobakhlou , B. Moghimi‐Dehkordi , N. Naderi , and F. Fadai . 2012. “Evaluation of Psychological Aspects Among Subtypes of Irritable Bowel Syndrome.” Indian Journal of Psychological Medicine 34, no. 2: 144–148. 10.4103/0253-7176.101780. 23162190 PMC3498777

[brb370513-bib-0024] Fazel, S. , and B. Runeson . 2020. “Suicide.” The New England Journal of Medicine 382, no. 3: 266–274. 10.1056/NEJMra1902944. 31940700 PMC7116087

[brb370513-bib-0025] Fishbain, D. A. , R. Cutler , H. L. Rosomoff , and R. S. Rosomoff . 1997. “Chronic Pain‐Associated Depression: Antecedent or Consequence of Chronic Pain? A Review.” Clinical Journal of Pain 13, no. 2: 116–137. 10.1097/00002508-199706000-00006. 9186019

[brb370513-bib-0026] Ford, A. C. , B. E. Lacy , L. A. Harris , E. M. M. Quigley , and P. Moayyedi . 2019. “Effect of Antidepressants and Psychological Therapies in Irritable Bowel Syndrome: An Updated Systematic Review and Meta‐Analysis.” The American Journal of Gastroenterology 114, no. 1: 21–39. 10.1038/s41395-018-0222-5. 30177784

[brb370513-bib-0027] Ford, A. C. , A. D. Sperber , M. Corsetti , and M. Camilleri . 2020. “Irritable Bowel Syndrome.” Lancet 396, no. 10263: 1675–1688. 10.1016/s0140-6736(20)31548-8. 33049223

[brb370513-bib-0028] Gralnek, I. M. , R. D. Hays , A. M. Kilbourne , L. Chang , and E. A. Mayer . 2004. “Racial Differences in the Impact of Irritable Bowel Syndrome on Health‐Related Quality of Life.” Journal of Clinical Gastroenterology 38, no. 9: 782–789. 10.1097/01.mcg.0000140190.65405.fb. 15365405

[brb370513-bib-0029] Greco, M. F. , C. Minelli , N. A. Sheehan , and J. R. Thompson . 2015. “Detecting Pleiotropy in Mendelian Randomisation Studies With Summary Data and a Continuous Outcome.” Statistics in Medicine 34, no. 21: 2926–2940. 10.1002/sim.6522. 25950993

[brb370513-bib-0030] Guo, Y. J. , C. H. Ho , S. C. Chen , S. S. Yang , H. M. Chiu , and K. H. Huang . 2010. “Lower Urinary Tract Symptoms in Women With Irritable Bowel Syndrome.” International Journal of Urology 17, no. 2: 175–181. 10.1111/j.1442-2042.2009.02442.x. 20088875

[brb370513-bib-0031] Hausteiner‐Wiehle, C. , and P. Henningsen . 2014. “Irritable Bowel Syndrome: Relations With Functional, Mental, and Somatoform Disorders.” World Journal of Gastroenterology 20, no. 20: 6024–6030. 10.3748/wjg.v20.i20.6024. 24876725 PMC4033442

[brb370513-bib-0032] Hubbard, C. S. , J. S. Labus , J. Bueller , et al. 2011. “Corticotropin‐Releasing Factor Receptor 1 Antagonist Alters Regional Activation and Effective Connectivity in an Emotional‐Arousal Circuit during Expectation of Abdominal Pain.” The Journal of Neuroscience: The Official Journal of the Society for Neuroscience 31, no. 35: 12491–12500. 10.1523/jneurosci.1860-11.2011. 21880911 PMC3399687

[brb370513-bib-0033] Jiang, C. , Y. Xu , S. Sharma , et al. 2019. “Association of Defecation Disorders With Suicidal Ideation in Young Adult With Chronic Abdominal Discomfort.” Journal of Affective Disorders 253: 308–311. 10.1016/j.jad.2019.05.004. 31078829

[brb370513-bib-0034] Jiang, C. , Y. Xu , S. Sharma , et al. 2019. “Psychosocial Factors Associated With Irritable Bowel Syndrome Development in Chinese College Freshmen.” Journal of Neurogastroenterology and Motility 25, no. 2: 233–240. 10.5056/jnm18028. 30870878 PMC6474708

[brb370513-bib-0035] Jung, H. K. , Y. H. Kim , J. Y. Park , et al. 2014. “Estimating the Burden of Irritable Bowel Syndrome: Analysis of a Nationwide Korean Database.” Journal of Neurogastroenterology and Motility 20, no. 2: 242–252. 10.5056/jnm.2014.20.2.242. 24840377 PMC4015204

[brb370513-bib-0036] Koloski, N. A. , N. J. Talley , and P. M. Boyce . 2000. “The Impact of Functional Gastrointestinal Disorders on Quality of Life.” The American Journal of Gastroenterology 95, no. 1: 67–71. 10.1111/j.1572-0241.2000.01735.x. 10638561

[brb370513-bib-0037] Kopczyńska, M. , Ł. Mokros , T. Pietras , and E. Małecka‐Panas . 2018. “Quality of Life and Depression in Patients With Irritable Bowel Syndrome.” Prz Gastroenterol 13, no. 2: 102–108. 10.5114/pg.2018.75819. 30002768 PMC6040097

[brb370513-bib-0038] Laird, K. T. , E. E. Tanner‐Smith , A. C. Russell , S. D. Hollon , and L. S. Walker . 2017. “Comparative Efficacy of Psychological Therapies for Improving Mental Health and Daily Functioning in Irritable Bowel Syndrome: A Systematic Review and Meta‐Analysis.” Clinical Psychology Review 51: 142–152. 10.1016/j.cpr.2016.11.001. 27870997

[brb370513-bib-0039] Lawlor, D. A. 2016. “Commentary: Two‐Sample Mendelian Randomization: Opportunities and Challenges.” International Journal of Epidemiology 45, no. 3: 908–915. 10.1093/ije/dyw127. 27427429 PMC5005949

[brb370513-bib-0040] Leite, G. , J. de Freitas Germano , W. Morales , et al. 2024. “Cytolethal Distending Toxin B Inoculation Leads to Distinct Gut Microtypes and Ibs‐D‐Like Microrna‐Mediated Gene Expression Changes in a Rodent Model.” Gut Microbes 16, no. 1:2293170. 10.1080/19490976.2023.2293170. 38108386 PMC10730147

[brb370513-bib-0041] Longarzo, M. , M. Quarantelli , M. Aiello , et al. 2017. “The Influence of Interoceptive Awareness on Functional Connectivity in Patients With Irritable Bowel Syndrome.” Brain Imaging and Behavior 11, no. 4: 1117–1128. 10.1007/s11682-016-9595-5. 27704405

[brb370513-bib-0042] Lovell, R. M. , and A. C. Ford . 2012. “Global Prevalence of and Risk Factors for Irritable Bowel Syndrome: A Meta‐Analysis.” Clinical Gastroenterology and Hepatology: The Official Clinical Practice Journal of the American Gastroenterological Association 10, no. 7: 712–721. e4. 10.1016/j.cgh.2012.02.029. 22426087

[brb370513-bib-0043] Magni, G. , S. Rigatti‐Luchini , F. Fracca , and H. Merskey . 1998. “Suicidality in Chronic Abdominal Pain: An Analysis of the Hispanic Health and Nutrition Examination Survey (Hhanes).” Pain 76, no. 1‐2: 137–144. 10.1016/s0304-3959(98)00035-9. 9696466

[brb370513-bib-0044] McIntyre, R. S. , M. Berk , E. Brietzke , et al. 2020. “Bipolar Disorders.” Lancet 396, no. 10265: 1841–1856. 10.1016/s0140-6736(20)31544-0. 33278937

[brb370513-bib-0045] McWilliams, L. A. , B. J. Cox , and M. W. Enns . 2003. “Mood and Anxiety Disorders Associated With Chronic Pain: An Examination in a Nationally Representative Sample.” Pain 106, no. 1‐2: 127–133. 10.1016/s0304-3959(03)00301-4. 14581119

[brb370513-bib-0046] Mearin, F. , X. Cortes , J. Mackinnon , J. Bertsch , J. Fortea , and J. Tack . 2014. “Economic and Quality‐of‐Life Burden of Moderate‐to‐Severe Irritable Bowel Syndrome With Constipation (Ibs‐C) in Spain: The Ibis‐C Study.” Value in Health: The Journal of the International Society for Pharmacoeconomics and Outcomes Research 17, no. 7: A365. 10.1016/j.jval.2014.08.811. 27200758

[brb370513-bib-0047] Midenfjord, I. , A. Polster , H. Sjövall , H. Törnblom , and M. Simrén . 2019. “Anxiety and Depression in Irritable Bowel Syndrome: Exploring the Interaction With Other Symptoms and Pathophysiology Using Multivariate Analyses.” Neurogastroenterology and Motility 31, no. 8:e13619. 10.1111/nmo.13619. 31056802

[brb370513-bib-0048] Miller, V. , L. Hopkins , and P. J. Whorwell . 2004. “Suicidal Ideation in Patients With Irritable Bowel Syndrome.” Clinical Gastroenterology and Hepatology: The Official Clinical Practice Journal of the American Gastroenterological Association 2, no. 12: 1064–1068. 10.1016/s1542-3565(04)00545-2. 15625650

[brb370513-bib-0049] Minocha, A. , D. Bollineni , W. D. Johnson , and W. C. Wigington . 2010. “Racial Differences in General Health, Suicidal Thoughts, Physical and Sexual Abuse in African‐Americans and Caucasians With Irritable Bowel Syndrome.” Southern Medical Journal 103, no. 8: 764–770. 10.1097/SMJ.0b013e3181e63653. 20622743

[brb370513-bib-0050] Mounier, N. , and Z. Kutalik . 2023. “Bias Correction for Inverse Variance Weighting Mendelian Randomization.” Genetic Epidemiology 47, no. 4: 314–331. 10.1002/gepi.22522. 37036286

[brb370513-bib-0051] Mullins, N. , J. Kang , A. I. Campos , et al. 2022. “Dissecting the Shared Genetic Architecture of Suicide Attempt, Psychiatric Disorders, and Known Risk Factors.” Biological Psychiatry 91, no. 3: 313–327. 10.1016/j.biopsych.2021.05.029. 34861974 PMC8851871

[brb370513-bib-0052] Nakao, M. , and E. Yano . 2006. “Somatic Symptoms for Predicting Depression: One‐Year Follow‐Up Study in Annual Health Examinations.” Psychiatry and Clinical Neurosciences 60, no. 2: 219–225. 10.1111/j.1440-1819.2006.01489.x. 16594947

[brb370513-bib-0053] Ng, Q. X. , Y. L. Lim , C. Y. L. Yaow , W. K. Ng , J. Thumboo , and T. M. Liew . 2023. “Effect of Probiotic Supplementation on Gut Microbiota in Patients With Major Depressive Disorders: A Systematic Review.” Nutrients 15, no. 6: 1351. 10.3390/nu15061351. 36986088 PMC10052013

[brb370513-bib-0054] Nock, M. K. , G. Borges , E. J. Bromet , et al. 2008. “Cross‐National Prevalence and Risk Factors for Suicidal Ideation, Plans and Attempts.” British Journal of Psychiatry 192, no. 2: 98–105. 10.1192/bjp.bp.107.040113. PMC2259024 18245022

[brb370513-bib-0055] Ressler, K. J. 2020. “Translating Across Circuits and Genetics Toward Progress in Fear‐ and Anxiety‐Related Disorders.” The American Journal of Psychiatry 177, no. 3: 214–222. 10.1176/appi.ajp.2020.20010055. 32114783 PMC7723454

[brb370513-bib-0056] Riedl, A. , M. Schmidtmann , A. Stengel , et al. 2008. “Somatic Comorbidities of Irritable Bowel Syndrome: A Systematic Analysis.” Journal of Psychosomatic Research 64, no. 6: 573–582. 10.1016/j.jpsychores.2008.02.021. 18501257

[brb370513-bib-0057] Saadh, M. J. , R. S. Pal , J. L. Arias‐Gonzáles , et al. 2023. “A Mendelian Randomization Analysis Investigates Causal Associations Between Inflammatory Bowel Diseases and Variable Risk Factors.” Nutrients 15, no. 5: 1202. 10.3390/nu15051202. 36904201 PMC10005338

[brb370513-bib-0058] Sekula, P. , M. F. Del Greco , C. Pattaro , and A. Köttgen . 2016. “Mendelian Randomization as an Approach to Assess Causality Using Observational Data.” Journal of the American Society of Nephrology 27, no. 11: 3253–3265. 10.1681/asn.2016010098. 27486138 PMC5084898

[brb370513-bib-0059] Smith, M. D. , A. Russell , and P. W. Hodges . 2008. “How Common Is Back Pain in Women With Gastrointestinal Problems?” Clinical Journal of Pain 24, no. 3: 199–203. 10.1097/AJP.0b013e31815d3601. 18287824

[brb370513-bib-0060] Smith, M. T. , R. R. Edwards , R. C. Robinson , and R. H. Dworkin . 2004. “Suicidal Ideation, Plans, and Attempts in Chronic Pain Patients: Factors Associated With Increased Risk.” Pain 111, no. 1‐2: 201–208. 10.1016/j.pain.2004.06.016. 15327824

[brb370513-bib-0061] Song, Z. , R. Yang , W. Wang , et al. 2021. “Association of Healthy Lifestyle Including a Healthy Sleep Pattern With Incident Type 2 Diabetes Mellitus Among Individuals With Hypertension.” Cardiovascular Diabetology 20, no. 1: 239. 10.1186/s12933-021-01434-z. 34922553 PMC8684653

[brb370513-bib-0062] Sperber, A. D. , Y. Atzmon , L. Neumann , et al. 1999. “Fibromyalgia in the Irritable Bowel Syndrome: Studies of Prevalence and Clinical Implications.” The American Journal of Gastroenterology 94, no. 12: 3541–3546. 10.1111/j.1572-0241.1999.01643.x. 10606316

[brb370513-bib-0063] Spiegel, B. , P. Schoenfeld , and B. Naliboff . 2007. “Systematic Review: The Prevalence of Suicidal Behaviour in Patients With Chronic Abdominal Pain and Irritable Bowel Syndrome.” Alimentary Pharmacology & Therapeutics 26, no. 2: 183–193. 10.1111/j.1365-2036.2007.03357.x. 17593064

[brb370513-bib-0064] Stern, P. 2021. “The Many Benefits of Healthy Sleep.” Science 374, no. 6567: 550–551. 10.1126/science.abm8113. 34709890

[brb370513-bib-0065] Tarar, Z. I. , U. Farooq , Y. Zafar , et al. 2023. “Burden of Anxiety and Depression Among Hospitalized Patients With Irritable Bowel Syndrome: A Nationwide Analysis.” Irish Journal of Medical Science 192, no. 5: 2159–2166. 10.1007/s11845-022-03258-6. 36593438

[brb370513-bib-0066] Turecki, G. , D. A. Brent , D. Gunnell , et al. 2019. “Suicide and Suicide Risk.” Nature reviews Disease primers 5, no. 1: 74. 10.1038/s41572-019-0121-0. 31649257

[brb370513-bib-0067] Tyagi, P. , M. Tasleem , S. Prakash , and G. Chouhan . 2020. “Intermingling of Gut Microbiota With Brain: Exploring the Role of Probiotics in Battle Against Depressive Disorders.” Food Research International 137: 109489. 10.1016/j.foodres.2020.109489. 33233143

[brb370513-bib-0068] Vasant, D. H. , P. A. Paine , C. J. Black , et al. 2021. “British Society of Gastroenterology Guidelines on the Management of Irritable Bowel Syndrome.” Gut 70, no. 7: 1214–1240. 10.1136/gutjnl-2021-324598. 33903147

[brb370513-bib-0069] Verbanck, M. , C. Y. Chen , B. Neale , and R. Do . 2018. “Detection of Widespread Horizontal Pleiotropy in Causal Relationships Inferred From Mendelian Randomization Between Complex Traits and Diseases.” Nature Genetics 50, no. 5: 693–698. 10.1038/s41588-018-0099-7. 29686387 PMC6083837

[brb370513-bib-0070] Whitehead, W. E. , O. Palsson , and K. R. Jones . 2002. “Systematic Review of the Comorbidity of Irritable Bowel Syndrome With Other Disorders: What Are the Causes and Implications?” Gastroenterology 122, no. 4: 1140–1156. 10.1053/gast.2002.32392. 11910364

[brb370513-bib-0071] Zhou, M. , Y. Fan , L. Xu , et al. 2023. “Microbiome and Tryptophan Metabolomics Analysis in Adolescent Depression: Roles of the Gut Microbiota in the Regulation of Tryptophan‐Derived Neurotransmitters and Behaviors in Human and Mice.” Microbiome 11, no. 1: 145. 10.1186/s40168-023-01589-9. 37386523 PMC10311725

[brb370513-bib-0072] Zhou, S. Y. , M. Gillilland 3rd , X. Wu , et al. 2018. “Fodmap Diet Modulates Visceral Nociception by Lipopolysaccharide‐Mediated Intestinal Inflammation and Barrier Dysfunction.” The Journal of Clinical Investigation 128, no. 1: 267–280. 10.1172/jci92390. 29202473 PMC5749529

