# Supplementary figures and images for: Causality Between Irritable Bowel Syndrome and Suicide Attempt: A Mendelian Randomization Study
Source: Brain Behav. 2025 May 5;15(5):e70513. doi: 10.1002/brb3.70513 (PMC12050657; doi:10.1002/brb3.70513)

IBS and suicide(ISGC)

Forward:
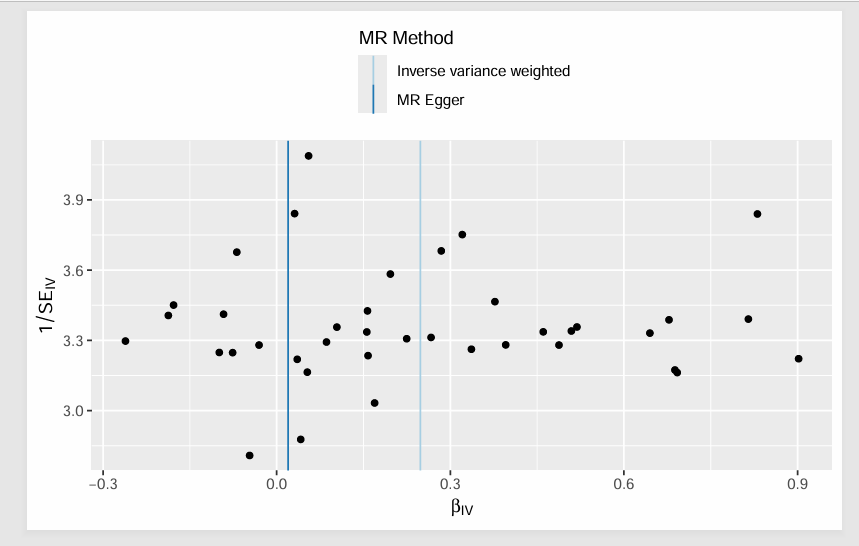

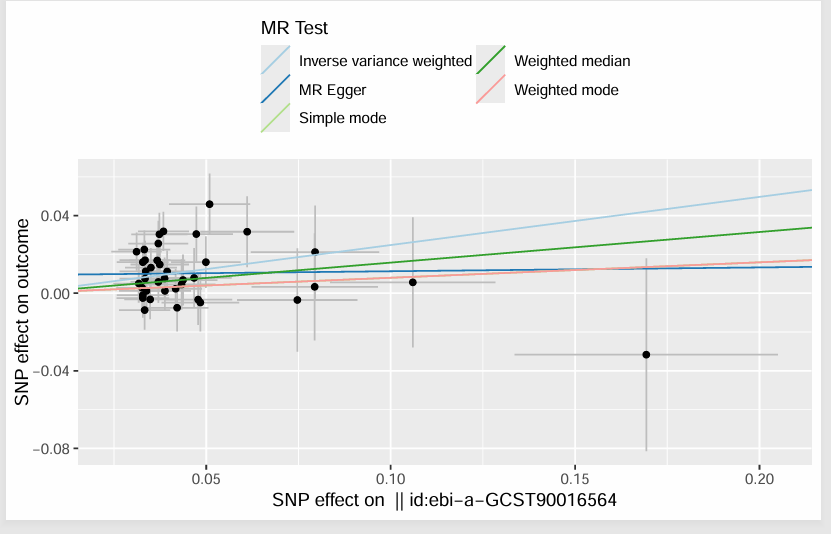

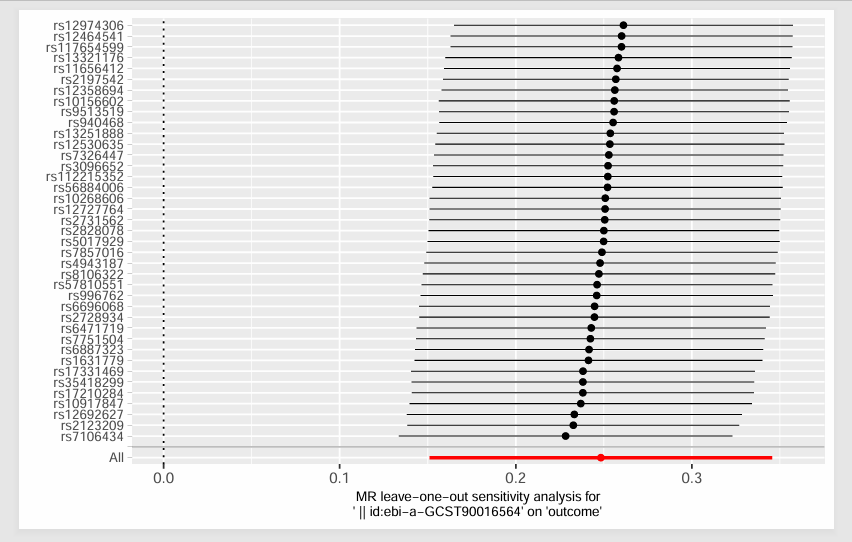


Reversr :


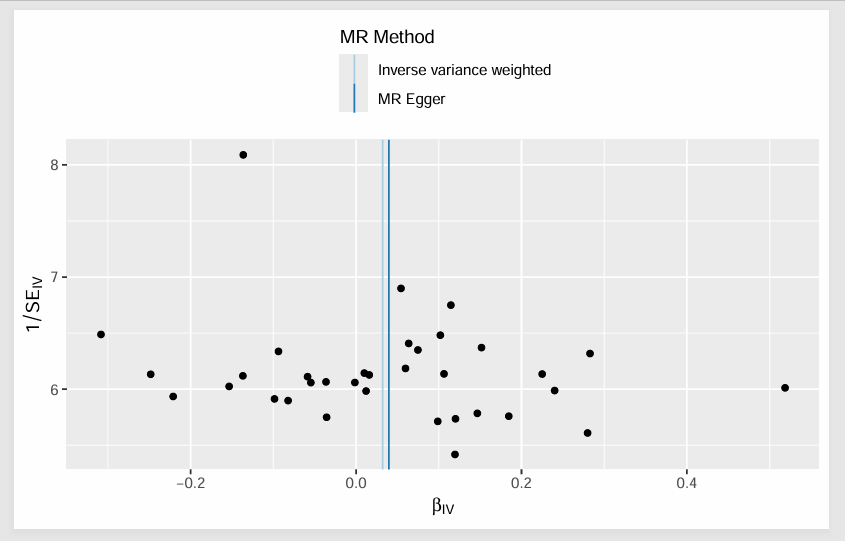

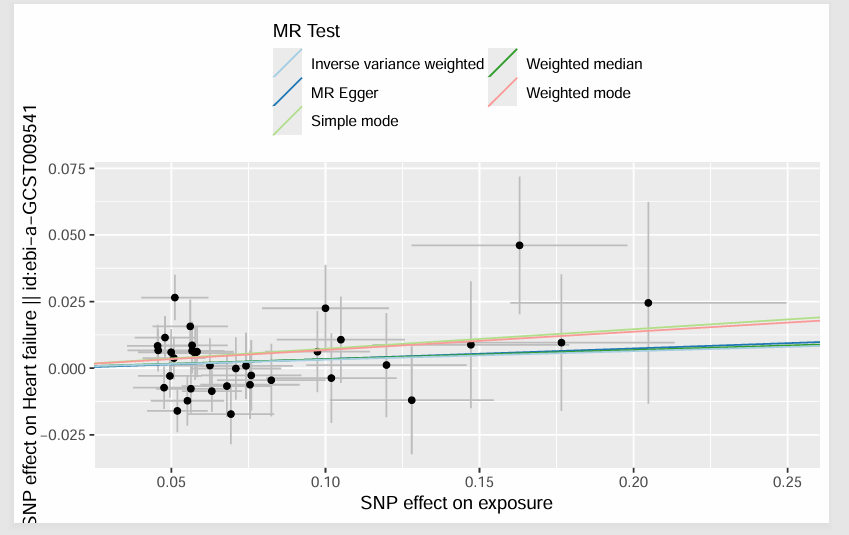

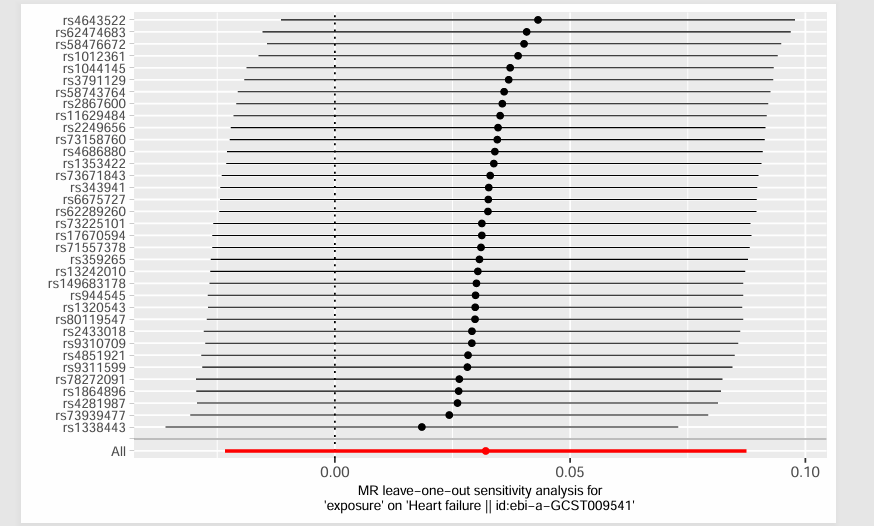


IBS and suicide(Finngen)

Forward:
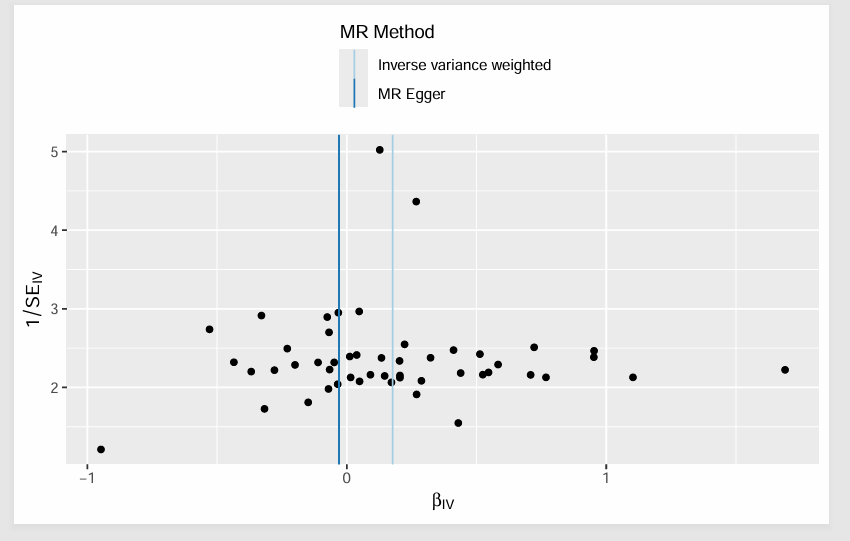


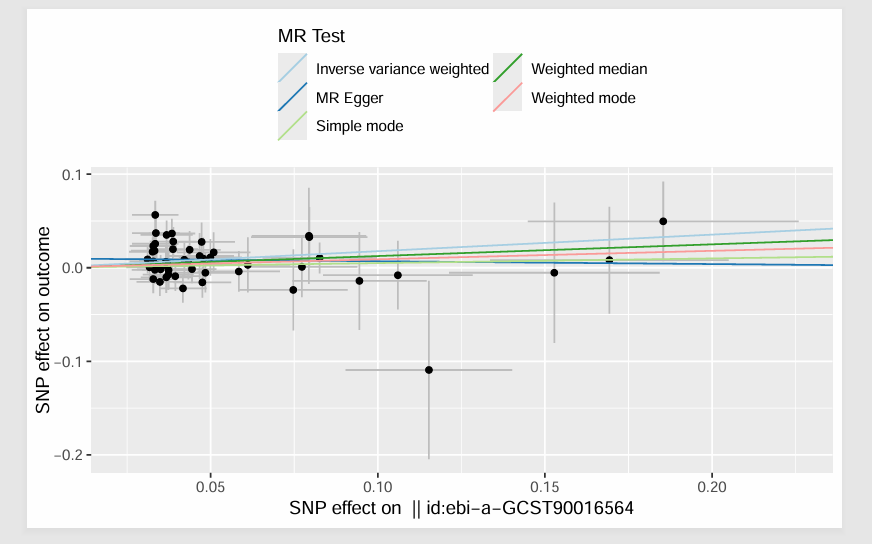

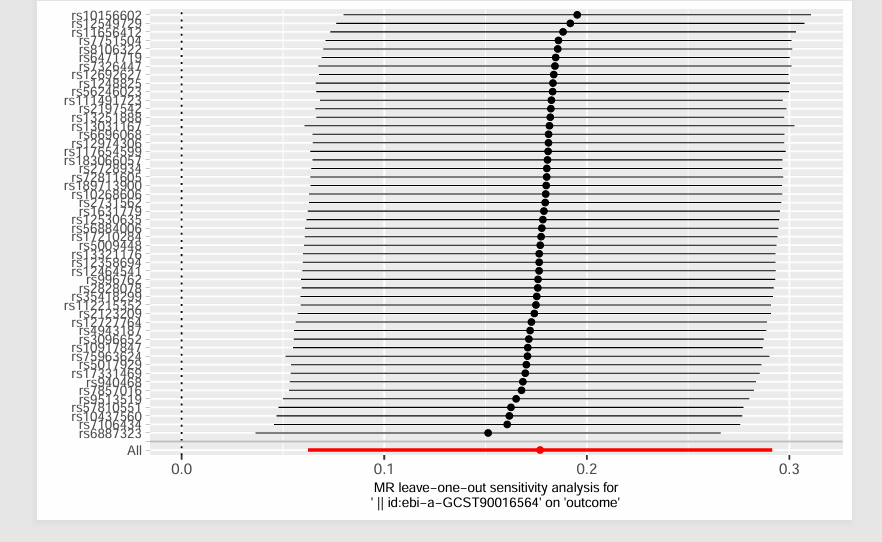


Reverse:


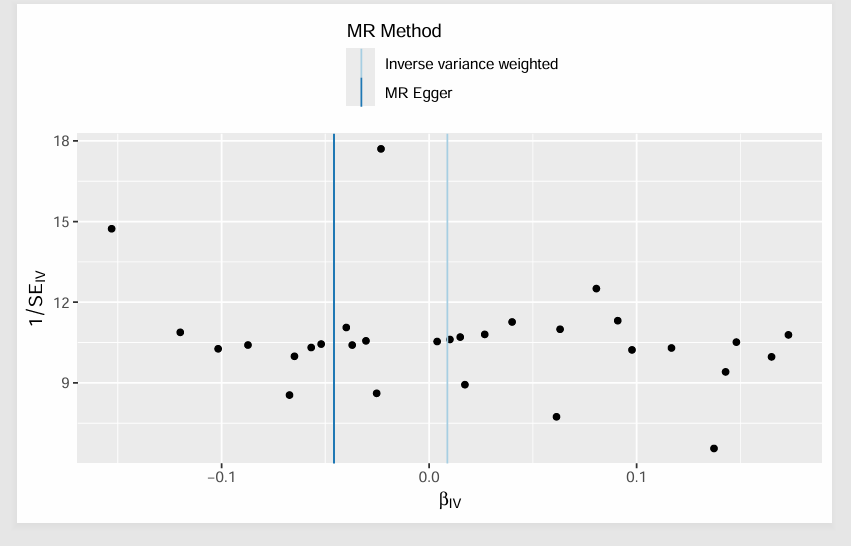

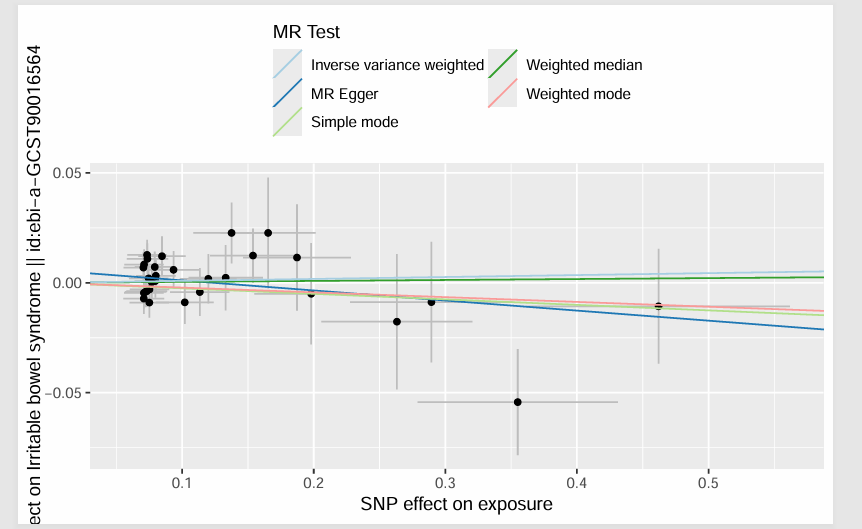

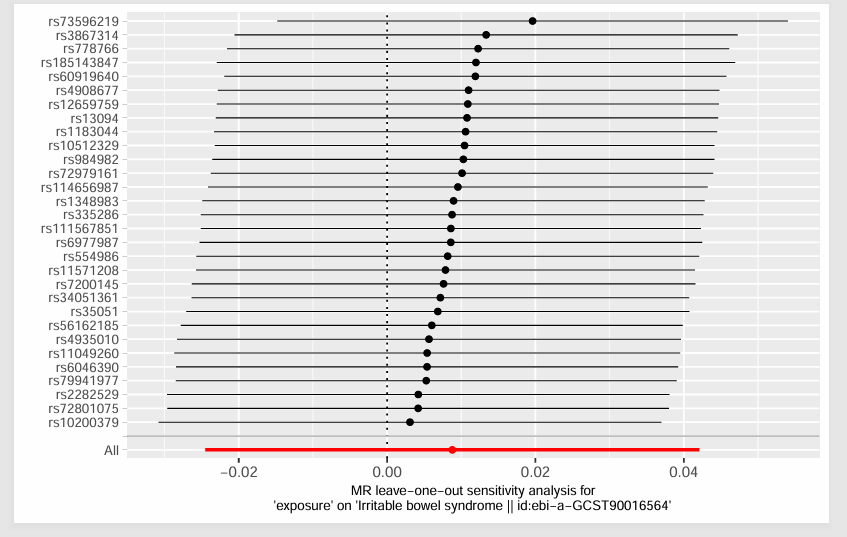

Supplement: Supplementary file 1 — Supporting Information [file BRB3-15-e70513-s001.docx]
